# Taskblaster: a generic framework for automated computational workflows

**DOI:** 10.1039/d5dd00097a

**Published:** 2025-08-01

**Authors:** Ask Hjorth Larsen, Mikael J. Kuisma, Tara M. Boland, Fredrik A. Nilsson, Kristian S. Thygesen

**Affiliations:** a CAMD, Computational Atomic-Scale Materials Design, Department of Physics, Technical University of Denmark Kgs. Lyngby 2800 Denmark asklarsen@gmail.com

## Abstract

We introduce Taskblaster, a generic and lightweight Python framework for composing, executing, and managing computational workflows with automated error handling. Taskblaster supports dynamic workflows including flow control using branches and iteration, making the system Turing complete. Taskblaster aims to promote modular designs, where workflows are composed of reusable sub-workflows, and to simplify data maintenance as projects evolve and change. We discuss the main design elements including workflow syntax, a storage model based on intuitively named tasks in a nested directory tree, and command-line tools to automate and control the execution of the tasks. Tasks are executed by worker processes that may run directly in a terminal or be submitted using a queueing system, allowing for task-specific resource control. We provide a library (ASR-lib) of workflows for common materials simulations employing the Atomic Simulation Environment and the GPAW electronic structure code, but Taskblaster can equally well be used with other computational codes.

## Abstract 2

Taskblaster is a powerful yet lightweight tool for dynamic, composable and reusable high-throughput computational workflows.

## Graphical abstract

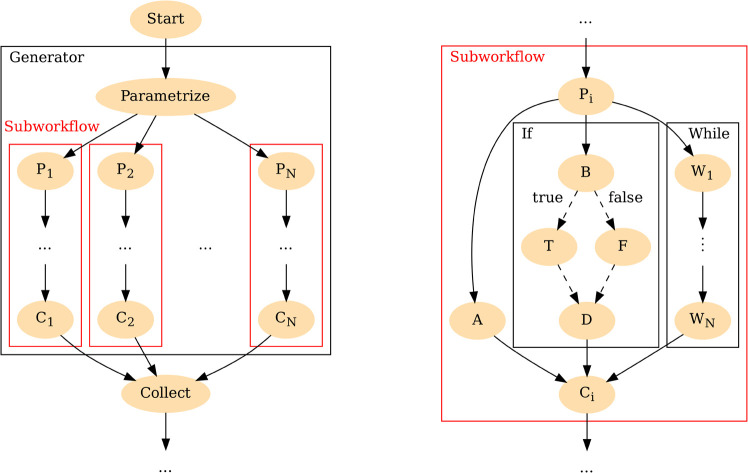

## 1. Introduction

In the forthcoming era of exascale computing, software tools to control and automate workflows will become indispensable for exploiting the computational resources effectively and harnessing the potential of big data science. Within the fields of computational chemistry and materials science, high-throughput computations are used more and more to identify optimal molecules or materials for different applications.^1–16^ The results of such studies are often stored in open databases^17–28^ to facilitate sharing and reuse of the data, not least for data analytics and machine learning purposes.^29–38^ For such an approach to be viable and successful, it is not only important to be able to efficiently execute many interdependent computational tasks with varying resource demands. One must also keep track of a sufficient amount of metadata to be able to track data provenance and allow the project's results to be reproduced and maintained over time.

On modern hardware, it is possible to create immense amounts of computational data in a short time. As a computational project progresses, both code and parameters will change: new computations must be done, code needs adaptation to support additional parameters, or underlying computational tools change. Many such changes cause computed results to be outdated with respect to the project code, and thus either the code must be updated or results must be patched or recomputed. Rather than computation time, the bottleneck quickly becomes the ability of researchers to maintain the generated data.

Here, we introduce Taskblaster – a Python framework executing computational workflows. Taskblaster (TB) workflows are defined using Python code. The workflow code defines a number of tasks, where each task encodes a future call to a Python function with particular inputs. Executing the workflow generates tasks and associated metadata as nodes of a directed acyclic graph (DAG) whose edges are the dependencies. Tasks can then be inspected or manipulated before configuring and launching parallel worker processes to run them. TB workflows support the use of branching, iteration, and dynamical generation of tasks, *i.e.*, generation of tasks depending dynamically on the outcome of other tasks.

Projects can customise certain behaviours using a plug-in mechanism. Most importantly, this includes how TB integrates with a parallel Python environment and how custom datatypes are encoded when saving inputs and outputs.

TB adds to a growing set of workflow management tools^39^ of which some originate from the materials science community.^20,40–49^ These tools differ in many aspects including data storage and representation (*e.g.* database servers *versus* local files), protocols used for determining data equivalence/conflicts (*e.g.* should a piece of calculated data be recalculated or is it consistent with the current inputs?), the type of logic operations supported, the handling of dynamic tasks, the way in which the resources are allocated on the compute system, and the way computational tasks are submitted.

Given the pivotal role that (big) data will be playing in the future, the importance of workflow control software cannot be understated and their continued development should be a priority alongside conventional simulation codes. In this regard, a heterogeneous set of workflow codes can lead to cross-fertilization and help identifying the most promising concepts and approaches.

Over the next sections we will discuss different aspects of Taskblaster and finally highlight features that we believe to be special. The article is structured as follows: Section 2 explains the overall design goals of TB. Section 3 describes features of TB in detail: tasks, static and dynamic workflows, data storage, configurable worker processes, input validation, and error handling. Section 4 describes ASR-lib, a library of TB workflows for atomistic high-throughput projects. Section 5 highlights specific notable features. Section 6 is a brief conclusion.

## 2. Why Taskblaster?

A computational project often starts with a single calculation in a single directory. The researcher adapts parameters and copies the input to a new directory to perform a related calculation. Additional copies appear as the project progresses. PhD students and postdocs develop collections of scripts to deal with the universal problem of how to adapt and make new calculations in this particular computational project, often with the ability to copy large numbers of files into intricate directory structures.

After the project, there will be an immense collection of scripts and utilities along with associated output data tailored to that specific project. Some data may be subtly outdated due to the gradual evolution of the code. In spite of high-quality publications, it may not be clear how to reproduce the results, even if both data and code still exist. Finally, the process for reproducing the data, should someone attempt to do so, is likely dependent on many manual steps since the original project evolved manually as well.

For a small project, that may not be an issue. However, projects with large valuable datasets are likely intended to be maintained and extended with new computations in the long term. Such projects will see generations of PhD students and postdocs making extensions and adaptations, and this requires a much higher standard for structure, transparency and documentation.

The goal of TB is to solve the problems described above. To that end, TB is designed to:

• Organize the project intuitively as a directory tree of meaningfully named tasks and workflows.

• Abstract the passage of data and files between tasks to avoid excessive coupling to filesystem paths or machine specific information.

• Work with large selections of tasks and achieve a high level of automation.

• Keep track of the task dependency tree in a way that makes it easy to see if any tasks are outdated with respect to the workflows that generated them.

Another goal of TB is to be easy to use. New projects should be easy enough to set up that researchers will not feel the temptation to develop large collections of custom project scripts, as discussed earlier. Furthermore, TB is a lightweight utility which requires no database services, network connections, or monitoring daemon processes, and works much the same whether on a laptop or a supercomputer.

However, there are also trade-offs: the desire to formally keep track of dependencies somewhat restricts the freedom to perform arbitrary processing inside workflows, since TB must be able to see any information passed between tasks in order to build the dependency tree and guarantee consistency. Hence, special constructs are needed for advanced workflow-level control flow, which otherwise might have been “ordinary” for-loops and if-statements.

## 3. Concepts and features

The typical way to use TB for a computational project is to connect *via* ssh to a supercomputer's login node and use the command-line interface while occasionally editing workflows or adding tasks. When starting a project, the first step is to initialise a repository. A repository is a directory on the disk with data related to the project. All data is kept as files inside this repository.

The next step is to define a main workflow. In principle, the main workflow defines every computation that will happen; in practice, it is gradually written as the project progresses. The main workflow can specify tasks, which are individual computations, and it can call other workflows, or subworkflows, which may likewise specify tasks and further subworkflows. A workflow also connects outputs from tasks to inputs of other tasks, defining the DAG.

Tasks and workflows are always assigned names. When subworkflows are nested, names are likewise nested. If a workflow named A defines a subworkflow, B, which defines a task, C, then the final name of that task will be 
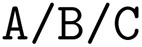
, and its files will be stored in 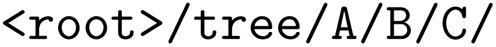, where 
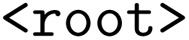
 is the root directory of the repository. The name of a task is therefore a global identifier for that task.

Operations on a repository are generally carried out using the TB command-line utilities. Examples are 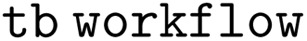 to run a workflow, 
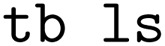
 to list tasks, and 
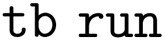
 to run tasks. Most commands take a list of task names as input. This can include shell wildcards (glob patterns) which make it easy to run operations on large selections of tasks. Once tasks are generated by a workflow, they can be run on the command-line or submitted *via* myqueue^43^ to an HPC job manager such as Slurm^50^ or Torque.^51^ TB runs tasks from worker processes that can be configured to pick up specific sets of tasks depending on the resources required. Once tasks run, they may succeed or fail, and workers keep picking up new tasks as long as there is time and there are available tasks that they are compatible with.

TB provides commands to remove tasks or “unrun” them. Removing a task deletes all its associated data and removes it from the DAG, whereas unrunning it only removes its output so that it can run again. Such commands work recursively on the dependency tree affecting all dependent tasks in topological order. Daily work often involves testing and revision of task implementations using many run/unrun cycles.

Furthermore, TB provides commands to list or view tasks in different levels of detail, to submit or manipulate workers, and to invoke actions that visualise or export data.

### 3.1. Tasks

In TB, a task represents the fundamental unit of computation within a workflow. Each task corresponds to a Python function and associated input specification, ideally in order to perform a single computational operation. The key constraint imposed on a TB task is that both the input and output must be serializable, *i.e.*, possible to store. This requirement ensures that tasks can be stored on the disk and later retrieved when a worker acquires the task.

The inputs of a task can be either specific objects such as numbers or arrays, or abstract references to the outputs of other tasks, or any nested structure (lists and dictionaries for example) involving both. Representing the input as a reference to a future output allows TB to construct large parts of the dependency tree without executing the tasks. The tree can thus be freely visualized and inspected, and the user can later choose to run tasks one at a time or in arbitrary groups. TB will automatically ensure that they run in topological order following the DAG. For example, if the user tries to run task B that depends on task A that has not yet been run, TB will first run task A before running task B.

**Fig. 1:**
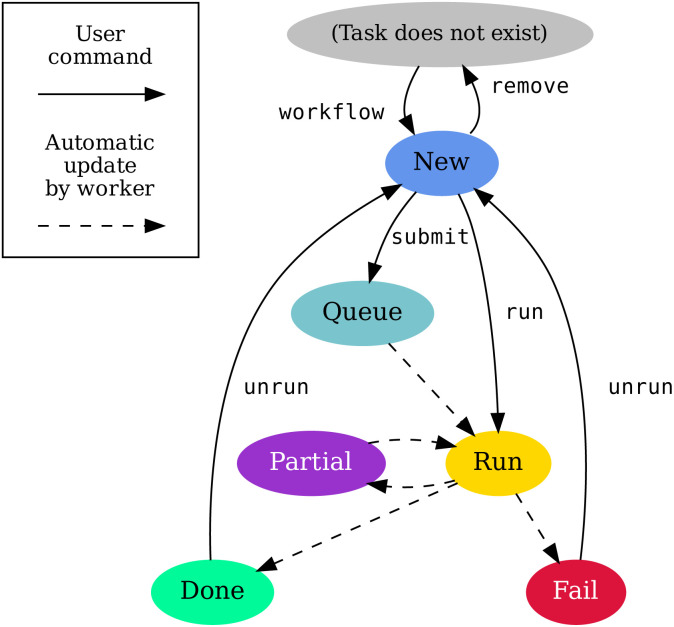
Most important commands (arrows) and how they affect the state of a task (ellipses). The workflow command generates a new task which the user can later submit, run, unrun, *etc.* Dashed arrows indicate that an update happens automatically as opposed to being controlled directly by the user.

Once tasks are generated by a workflow, they can be manipulated using the command-line interface. A newly generated task starts in the “new” state, which means it is eligible to run once all its inputs are available. Issuing a 
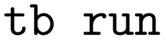
 command will change its state to “run”, after which it may change to “done” or “fail” depending on success. Fig. 1 shows the most important task states and how tasks transition between them *via* commands. Tasks can also go into a “partial” state in connection with error handling, or a “queue” state to signify that it may be picked up by a worker.

Some tasks may require runtime information about the machine or parallelization that is neither a global constant nor a proper input parameter. TB provides a Python decorator to inject such information into tasks without affecting (and hence invalidating upon change) the stored input. This includes MPI communicators and hardware flags such as whether to use a GPU. In addition, TB provides syntax and command-line tools to tag tasks according to which computational resources they need.

Tasks can be equipped with error handlers that can run in multiple stages. The special “partial” state is used when a task did not succeed, but may yet succeed if it has error handlers that did not run yet, and might recover from the failure.

### 3.2. Static workflow constructs

Workflows are prescriptions for generating tasks and subworkflows, and they are defined using a number of different syntactic constructs. The simplest construct is the statically defined task: running the workflow produces that task, but each such task must be hardcoded on that workflow. Similarly, a subworkflow is a static declaration to run another workflow as part of this one.

**Fig. 2:**
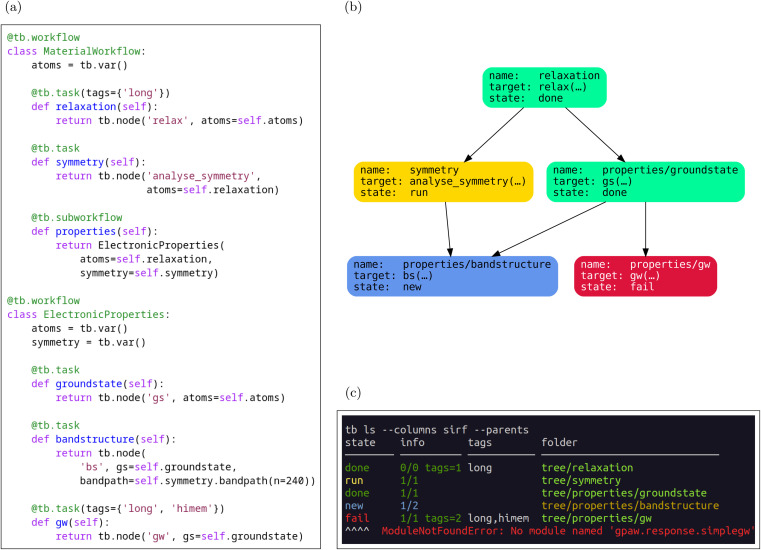
(a) Workflow with two tasks and a subworkflow with a further three tasks. This example is based on real computational workflows, but with complexity and number of parameters greatly reduced. 
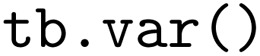
 defines input variables for the workflows. Each 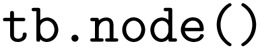 call is a specification of a node to the DAG whereas the 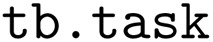 decorator equips it with metadata such as tags. Full examples including how to initialize the repository and workflow can be found in the documentation.^52^ (b) DAG generated by the workflow and visualised using the 
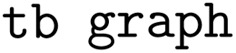
 command. At the workflow level, passing a task such as 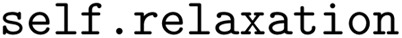 to 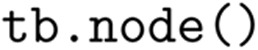 inside the symmetry task creates a dependency. Hence the symmetry task will depend on the relaxation task. Running the workflow adds (or updates) its tasks to the tree (c) from which they can be further processed. The tree is visualised with the 
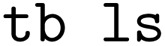
 command, where some of the tasks already ran. The “info” column shows number of done/total dependencies of each task: the bandstructure task is waiting for the symmetry task to finish. Note how the names of the methods in the workflow class determine the directory nesting, and how the “properties” subworkflow creates an additional nested directory for its tasks.

Fig. 2a shows an example of how a static workflow is defined using Python syntax. The workflow is a class; each task is a decorated method to return a node for the DAG on Fig. 2b. The 
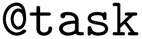
 decorator can be used to specify rules for computational resources and error handling. Note how the workflow specifies the routing between tasks, *i.e.*, which outputs from which tasks to connect to which inputs of others. The inputs must match the call signature of the target function: the relaxation job implies that there is a function named relax which takes an input named atoms. These inputs need not exist yet when the workflow runs; instead, entities like 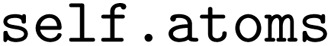 or 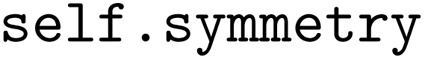 are future references which specify that the parameter is to be loaded and passed to the target function when a task runs. Additionally, the syntax supports indexing, attribute access, and method calls into the outputs of other tasks. For example, the expression 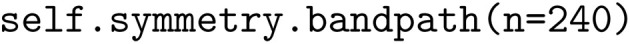 under the 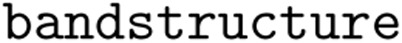 specification does not actually perform a function call, but rather saves an encoded representation of that call so that it can be evaluated when the corresponding task runs.

Running the workflow builds the tree of tasks. Fig. 2c shows a screenshot produced by the 
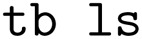
 command listing the state of tasks, their dependencies (done/total), requested resources, and location in the directory tree.

The workflow syntax bears similarities to the Workflow Definition Language OpenWDL^53,54^ in terms of subworkflows as well as routing of inputs and outputs. The TB syntax, being written in Python, provides convenience for projects that are written in Python and can simplify object serialisation.

### 3.3. Dynamical workflow constructs

**Fig. 3:**
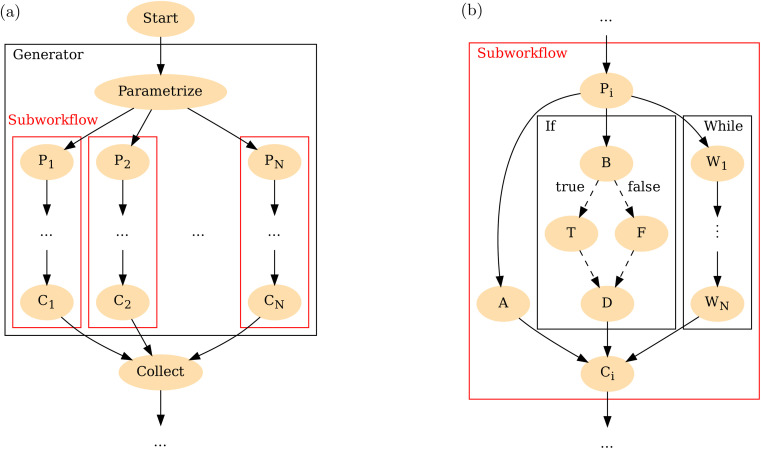
Example of a full workflow illustrating multiple features. Ellipses represent tasks. (a) Top-level workflow which uses a generator to dynamically parametrize over an input dataset, applies a subworkflow on each element P_1_⋯P_*N*_, and collects results C_1_⋯C_*N*_. (b) An example of a subworkflow which includes statically defined tasks as well as an If-statement and a While-loop producing tasks W_1_⋯W_*N*_. Dashed lines within the If-statement indicate that only one of the two tasks T or F is created. A, B and D are arbitrary static tasks.

Support for dynamical workflows, that is, workflows in which the number or type of tasks are decided dynamically depending on calculated data, rather than being coded statically, is provided through additional constructs that can be categorized as “one-to-many” (parametrisation), “many-to-one” (collection), and conditional branching. Fig. 3 shows an example of how these can be used in a full workflow.

#### 3.3.1. Parametrization and collection

To apply a workflow to many elements in a dataset of variable size, one can use a generator. A generator is a construct which, as it runs, has access to the physical output of its input tasks and may generate any number of workflows or tasks using that. Fig. 3a shows a typical workflow in materials physics which starts with an input database of crystal structures and uses a generator to apply a materials workflow to each. Since generators are dynamic, a workflow cannot directly add new tasks that depend on tasks from generators: it is not known statically how many tasks there will be or what their names will be. Instead, the Generator can be equipped with fixed points, which are special tasks that can access all or some of the generated tasks according to a rule, for example, all resulting materials with Fe in them. The calling workflow then uses the fixed points to access groups of tasks within the generator and pass them on to other tasks or subworkflows outside it. On Fig. 3a, the collect task involves a fixed point assembling information from each subworkflow.

#### 3.3.2. Control flow

**Fig. 4:**
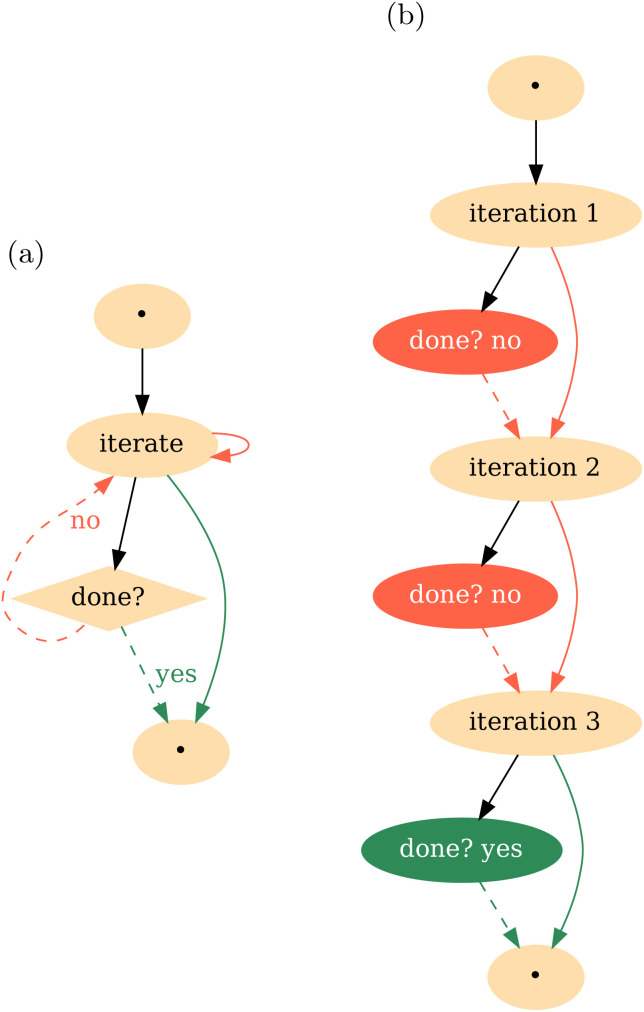
(a) Control flow for a “do-while” loop workflow. The iterate task directly connects to itself since one iteration's input is the previous iteration's output, whereas the decision of whether to terminate the loop is done by a separate task. Solid lines denote ordinary dependencies whereas dashed lines denote decisions made at the workflow level. The initial and final tasks, labelled “•”, can be static. Other tasks and workflows can be defined that depend on those even though the loop did not run yet. (b) Example of DAG generated by running the loop.

Fig. 3b highlights one of the subworkflows presented on Fig. 3a. It shows two distinct conditional constructs, namely branching with an if-statement and a while loop. A key concept in understanding TB workflows is the distinction between the control flow and the DAG. The control flow is a sequence of processing steps which allows loops and branches. The control flow can be understood as a possibly cyclic directed graph, and different DAGs can be generated by different executions of the control flow depending on concrete input data. To illustrate this point further, we show these two representations on Fig. 4. Fig. 4a represents the workflow which will generate the DAG shown on Fig. 4b. Each arrow on Fig. 4a corresponds to one or many arrows on Fig. 4b. In contrast to static routings, where each task has hardcoded dependencies, with branching, the routings need to be dynamic. For example, in Fig. 4a the input to the iterate task comes either from the initial task, labelled “•”, or from the iterate task itself, depending on whether control flow is at the first iteration or not. On the other hand, once the DAG (Fig. 4b) is constructed, it remains constant, indicating the final dependencies of each task.

### 3.4. Data storage

Running a workflow results in the creation of tasks that have yet to run. The tasks are stored in a SQLite database called the registry. The information stored is the import path of the function to be called along with an input specification which can contain any JSON-encodable object including a reference to another task. TB builds the dependency tree by inspecting these inputs and saves all the metadata in the registry for efficient retrieval.

Once a task runs, it is assigned a directory on the disk where its outputs are stored along with its input specification as JSON. This provides a level of redundancy which allows the registry to be reconstructed in case of corruption due to power outages, bugs, or user errors. Tasks may also leave arbitrary files in their directory, which is useful for storing larger outputs from computations that it would be inefficient to encode using JSON, or which are not useful to represent directly as Python objects. Tasks can return path objects pointing to files they generated in order for other tasks to access those paths *via* their input. TB takes care of storing these paths in a way that is robust with respect to moving a repository. TB automatically ensures that the path points to the correct location when used in subsequent tasks, although they run in a different directory.

To save and load Python objects, TB must be able to serialize those objects. TB itself supports only basic objects. A custom JSON encoder can be specified *via* a plug-in: the TB repository is configured to point to a special plug-in module which can specify a custom JSON encoder. For example ASR-lib uses this to integrate with the ASE encoder and hence supports commonly used objects including numpy arrays and ASE Atoms objects. Custom classes written by a user can also be supported by adding an encoding hook.

### 3.5. Workers and resources

The simplest way to run a task is to issue a 
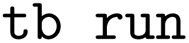
 command specifying one or more tasks or directories with tasks. This launches a worker to run the selected tasks. The worker automatically resolves each task's dependency tree and executes, to the extent possible, all required tasks in topological order.

The user can configure multiple kinds of workers in a special configuration file. This facilitates specification of computational resources and encompasses number of cores, Slurm partition, walltime, and more. Multiple configured workers can then be submitted simultaneously and with a single command. Submitting a worker is, in principle, no different from submitting a 
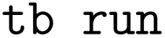
 command with specific settings. TB submits workers *via* myqueue.^43^

Tasks and workers can be equipped with configurable resource tags, like the relaxation task on Fig. 2a which has the “long” tag. Workers only pick up tasks with matching tags. For example, there can be one kind of worker intended for lightweight processing, another worker for heavy computations, and a third worker for computations that require particular hardware such as a GPU. Multiple “subworkers” can be configured to run inside a single HPC job. Doing so can allow better sharing of resources when tasks require fewer resources than a whole node.

### 3.6. Changing and validating inputs

A user rarely knows the best computation parameters at the beginning of a project. A large amount of trial and error is often necessary to establish good parameters before scaling a project to large datasets. In TB, such trial and error would normally be done by successively unrunning and rerunning tasks. However, later on, once large amounts of production data exists, it could be expensive to rerun tasks any time anything changes. Also, some changes—such as minor tweaking of computational settings or refactoring of input parameter to tasks—do not always warrant unrunning and rerunning the task since the changes do not affect the end result.

To handle situations such as these, TB alerts the user to “conflicts” when the input data changes in the workflow. When changing a parameter, a user would adapt the workflow and then rerun it. This generates all the same tasks that already exist, but TB detects that an input has changed. The affected tasks will then be marked as conflicted, which “freezes” them along with every task that depends on them, preventing those tasks from running. The user must either unrun them or mark the conflicts as “resolved” in order to unfreeze the frozen tasks, indicating that the conflict does not affect data integrity. A resolved conflict is simply a way to tell the workflow that the task's results are to be kept as they are even though the inputs are different. Both original and new (conflicting) inputs are saved. TB can also show a diff highlighting the specific changes in input parameters.

### 3.7. Error handling

Numerical simulations often fail in ways that cannot be predicted before the computations run. Structure optimizations may not converge due to inaccurate forces or bad conditioning, DFT calculations may not converge, any simulation may run out of memory. Across very large datasets, any error, no matter how improbable, may arise due simply to the number of systems and diversity of inputs.

TB provides a system, the warden, for solving this problem. If a task fails, the user can implement an error handler and modify the workflow to equip the task with the handler. The handler can execute any code to recover from the error. Often this means rerunning the target function of the task with modified inputs or using a checkpoint. Other tasks of the same type will automatically have the error handler as well. Multiple such handlers can execute in succession or in response to different errors, interacting with the warden using a particular programming interface.

Overall, the execution of error handlers is considered part of a task (as opposed to being represented as a succession of different tasks) and corresponds closely to typical “try/except” exception handling as supported in modern programming languages. Error handlers do not change the task inputs as stored, as that would cause a conflict. Instead, they have access to call the target function with a modified set of inputs after the original function fails.

## 4. ASR-lib

The Atomic Simulation Recipes Library (ASR-lib) is a library of TB workflows for materials and molecular simulations. Atomistic computational backends, such as density functional theory (DFT) codes or (machine learning) interatomic potentials, can be called *via* the calculator interface of the Atomic Simulation Environment (ASE).^55^ ASR-lib and TB serve as a more scalable and reusable replacement for the previous ASR project^56^ which encompasses a simulation code library as well as workflow management features for legacy projects.

The workflows in ASR-lib are written in a general style and can be used for any type of material, independent of dimensionality and composition. Consequently, the workflows in ASR-lib can be used as initial templates when producing more project specific workflows. Currently, ASR-lib contains workflows for many different operations/calculations, and is continuously being developed. Most of these employ the GPAW^57,58^ electronic structure code as a calculator, but it is straightforward to generalise to other types of calculators as long as they have a Python interface, *e.g. via* ASE. Below, we mention a few examples of workflows in ASR-lib, highlighting features that are enabled by TB.

For example, the GW and Phonon workflows utilize generators to generate *q*-point and displacement parallelisations at the task level. The structure relaxation workflow contains several branches and a while loop related to searching for the lowest energy magnetic configuration. The crystal defect workflows can dynamically generate various types of point defects using a generator and subsequently proceed with nested generators to classify their properties (formation energy, charge transition levels, *etc.*) by means of DFT calculations. ASR-lib also contains examples of large-scale data processing of existing trees, like evaluation of the energy above the convex hull. These so-called “from tree” methods can be used to collect data from TB repositories, perform analysis, and spawn projects with a new focus.

ASR-lib is currently used in a number of ongoing high-throughput projects related to 2D materials and point defects. In addition to ASR-lib, TB has also been independently used for workflows based on the FHI-Aims code.^59^

## 5. Characteristic features

TB is written in Python and released under the GNU General Public License, version 3 or later. Its only software requirement aside from the Python standard library is the lightweight click package for command-line interface support. In practice, TB will normally be used together with myqueue and an HPC queueing system like Slurm^50^ or Torque.^51^ Code and documentation are available online.^60,61^

Other distinguishing features of TB are:

• Low infrastructure requirements: TB runs in any Python environment and does not require persistent network connections or database processes.

• Intuitive data storage: workflows and tasks are organised in a directory tree where nested subdirectories serve as namespaces.

• Automatic I/O: TB automates the loading and saving of Python objects as inputs and outputs and works to reduce filesystem path clutter throughout the code.

• General purpose: TB is a generic workflow tool and is not linked to any domain-specific simulation software.

• Plug-ins: users can facilitate work with domain-specific simulation software by writing a plug-in as in the case of ASR-lib.

• Configurable computational resources: tasks are executed by configurable worker processes, where each worker process can run any set of tasks. The logical division of a workflow into tasks is independent of the number or type of actual HPC jobs that run the tasks. Additionally, machine-specific configuration can be kept separate from the main project code.

In general, the top-level workflow encodes every computation that is going to happen. The command-line interface cannot itself add computations or change any result. It only provides a way for the user to choose what, when, and how to run. When running a workflow, TB eagerly adds as many tasks as possible to the DAG without executing any of them. This allows the user to “see into the future” and better assess the required resources, or to experiment with a subset of tasks using the characteristic “run/unrun” pattern. TB can generate parts of the DAG that depend on a dynamical workflow, even though the workflow did not run yet. This is possible because TB can use fixed-point tasks on the dynamic workflow to infer the existence of subsequent tasks. The fragments are then connected to a final DAG once the dynamical workflow runs.

## 6. Conclusion

We have described the most important concepts in Taskblaster, how tasks are generated and organised, and how to run calculations.

TB aims to bridge the gap from small to large projects: it can act as a simple tool to automate processing steps locally on a laptop, or used in large projects that needs to scale and adapt over time. Major design features of TB are: intuitive organisation of data using a directory tree, a usage model which minimises infrastructure requirements by emphasising local data storage and interactive work in a terminal, avoiding the need for heavy-weight database connections, while keeping a strict representation of task dependencies as a persistent DAG.

We have found that this combination facilitates an efficient “unrun/rerun”-based approach to practical experimentation, which is often required in the development phase of new computational projects.

Most core design elements of TB are unlikely to change in their main structure, so future TB development will increasingly focus on smaller improvements to user experience, helper functionality for data migration and other tools that prove useful as the projects using TB mature further.

## Conflicts of interest

There are no conflicts to declare.

## Data Availability

The paper presents the Taskblaster project. Taskblaster code and version history are publically available. The contents of the manuscript correspond to taskblaster-0.2. The release source code can be found on zenodo: https://doi.org/10.5281/zenodo.16363818. The release is available on pypi: https://pypi.org/project/taskblaster/0.2/. The source code can be found on the Gitlab repository: https://gitlab.com/taskblaster/taskblaster/. The taskblaster-0.2 release corresponds to the git commit: eadbf847f60d5474d7bc4957be941c40846f7ac0 (tag: 0.2). The manuscript refers to ASR-lib without directly presenting any specific data from the project. However the code is publically available on Gitlab as well: https://gitlab.com/asr-dev/asr-lib. There is no further code or data associated with the published work.

## References

[cit1] Greeley J., Jaramillo T. F., Bonde J., Chorkendorff I., Nørskov J. K. (2006). Nat. Mater..

[cit2] Madsen G. K. (2006). J. Am. Chem. Soc..

[cit3] Curtarolo S. (2013). et al.. Nat. Mater..

[cit4] Kirklin S., Meredig B., Wolverton C. (2013). Adv. Energy Mater..

[cit5] Ørnsø K. B., Garcia-Lastra J. M., Thygesen K. S. (2013). Phys. Chem. Chem. Phys..

[cit6] Zhang Z. (2019). et al.. ACS Omega.

[cit7] Chen W. (2016). et al.. J. Mater. Chem. C.

[cit8] Hachmann J., Olivares-Amaya R., Atahan-Evrenk S., Amador-Bedolla C., Sánchez-Carrera R. S., Gold-Parker A., Vogt L., Brockway A. M., Aspuru-Guzik A. (2011). J. Phys. Chem. Lett..

[cit9] Bhattacharya S., Madsen G. K. (2015). Phys. Rev. B: Condens. Matter Mater. Phys..

[cit10] Castelli I. E. (2012). et al.. Energy Environ. Sci..

[cit11] Hautier G., Miglio A., Ceder G., Rignanese G.-M., Gonze X. (2013). Nat. Commun..

[cit12] Yu L., Zunger A. (2012). Phys. Rev. Lett..

[cit13] Kuhar K., Pandey M., Thygesen K. S., Jacobsen K. W. (2018). ACS Energy Lett..

[cit14] Aykol M., Kim S., Hegde V. I., Snydacker D., Lu Z., Hao S., Kirklin S., Morgan D., Wolverton C. (2016). Nat. Commun..

[cit15] Mounet N., Gibertini M., Schwaller P., Campi D., Merkys A., Marrazzo A., Sohier T., Castelli I. E., Cepellotti A., Pizzi G., Marzari N. (2018). Nat. Nanotechnol..

[cit16] Chen L.-Q., Chen L.-D., Kalinin S. V., Klimeck G., Kumar S. K., Neugebauer J., Terasaki I. (2015). npj Comput. Mater..

[cit17] Thygesen K. S., Jacobsen K. W. (2016). Science.

[cit18] Saal J. E., Kirklin S., Aykol M., Meredig B., Wolverton C. (2013). JOM.

[cit19] Jain A. (2013). et al.. APL Mater..

[cit20] Curtarolo S., Setyawan W., Hart G. L., Jahnatek M., Chepulskii R. V., Taylor R. H., Wang S., Xue J., Yang K., Levy O. (2012). et al.. Comput. Mater. Sci..

[cit21] Draxl C., Scheffler M. (2019). J. Phys Mater..

[cit22] Haastrup S., Strange M., Pandey M., Deilmann T., Schmidt P. S., Hinsche N. F., Gjerding M. N., Torelli D., Larsen P. M., Riis-Jensen A. C. (2018). et al.. 2D Mater..

[cit23] Borysov S. S., Geilhufe R. M., Balatsky A. V. (2017). PLoS One.

[cit24] Winther K. T., Hoffmann M. J., Boes J. R., Mamun O., Bajdich M., Bligaard T. (2019). Sci. Data.

[cit25] Talirz L., Kumbhar S., Passaro E., Yakutovich A. V., Granata V., Gargiulo F., Borelli M., Uhrin M., Huber S. P., Zoupanos S. (2020). et al.. Sci. Data.

[cit26] Armiento R. , Machine Learning Meets Quantum Physics, 2020, pp. 377–395

[cit27] Himanen L., Geurts A., Foster A. S., Rinke P. (2019). Adv. Sci..

[cit28] Gjerding M. N., Taghizadeh A., Rasmussen A., Ali S., Bertoldo F., Deilmann T., Knøsgaard N. R., Kruse M., Larsen A. H., Manti S., Pedersen T. G., Petralanda U., Skovhus T., Svendsen M. K., Mortensen J. J., Olsen T., Thygesen K. S. (2021). 2D Materials.

[cit29] Rupp M., Tkatchenko A., Müller K.-R., Von Lilienfeld O. A. (2012). Phys. Rev. Lett..

[cit30] Lee J., Seko A., Shitara K., Nakayama K., Tanaka I. (2016). Phys. Rev. B.

[cit31] Xie T., Grossman J. C. (2018). Phys. Rev. Lett..

[cit32] Ghiringhelli L. M., Vybiral J., Levchenko S. V., Draxl C., Scheffler M. (2015). Phys. Rev. Lett..

[cit33] Jørgensen P. B., del Río E. G., Schmidt M. N., Jacobsen K. W. (2019). Phys. Rev. B.

[cit34] Ghosh K., Stuke A., Todorović M., Jørgensen P. B., Schmidt M. N., Vehtari A., Rinke P. (2019). Adv. Sci..

[cit35] Deringer V. L., Csanyi G. (2017). Phys. Rev. B.

[cit36] Lorenz S., Gross A., Scheffler M. (2004). Chem. Phys. Lett..

[cit37] Behler J., Parrinello M. (2007). Phys. Rev. Lett..

[cit38] Artrith N., Urban A. (2016). Comput. Mater. Sci..

[cit39] Existing Workflow Systems, https://s.apache.org/existing-workflow-systems, Accessed 2025-06-06, Updated 2025-03-17

[cit40] Jain A. (2015). et al.. Concurrency Comput..

[cit41] Pizzi G., Cepellotti A., Sabatini R., Marzari N., Kozinsky B. (2016). Comput. Mater. Sci..

[cit42] Mathew K., Montoya J. H., Faghaninia A., Dwarakanath S., Aykol M., Tang H., Chu I.-h., Smidt T., Bocklund B., Horton M. (2017). et al.. Comput. Mater. Sci..

[cit43] Mortensen J. J., Gjerding M., Thygesen K. (2020). J. Open Source Softw..

[cit44] Rosen A. S., Gallant M., George J., Riebesell J., Sahasrabuddhe H., Shen J.-X., Wen M., Evans M. L., Petretto G., Waroquiers D. (2024). et al.. J. Open Source Softw..

[cit45] Janssen J., Surendralal S., Lysogorskiy Y., Todorova M., Hickel T., Drautz R., Neugebauer J. (2019). Comput. Mater. Sci..

[cit46] Armiento R. , in Database-Driven High-Throughput Calculations and Machine Learning Models for Materials Design, ed. K. T. Schütt, S. Chmiela, O. A. von Lilienfeld, A. Tkatchenko, K. Tsuda and K.-R. Müller, Springer International Publishing, Cham, 2020, pp. 377–395

[cit47] Sjølin B. H., Hansen W. S., Morin-Martinez A. A., Petersen M. H., Rieger L. H., Vegge T., García-Lastra J. M., Castelli I. E. (2024). Digital Discovery.

[cit48] Atwi R., Bliss M., Makeev M., Rajput N. N. (2022). Sci. Rep..

[cit49] https://github.com/wolverton-research-group/qmpy

[cit50] Jette M. A. and Wickberg T., Job Scheduling Strategies for Parallel Processing, Cham, 2023, pp. 3–23

[cit51] Staples G. , Proceedings of the 2006 ACM/IEEE Conference on Supercomputing, New York, NY, USA, 2006, p. 8es

[cit52] Taskblaster tutorials, https://taskblaster.readthedocs.io/en/latest/tutorial/module.html

[cit53] https://openwdl.org/

[cit54] Voss K. , Van Der Auwera G. and Gentry J., Full-stack genomics pipelining with GATK4 + WDL + Cromwell [version 1; not peer reviewed], F1000Research, 2017, vol. 6, p. 1381, 10.7490/f1000research.1114634.1

[cit55] Larsen A. H., Mortensen J. J., Blomqvist J., Castelli I. E., Christensen R., Dułak M., Friis J., Groves M. N., Hammer B., Hargus C. (2017). et al.. J. Phys.: Condens. Matter.

[cit56] Gjerding M., Skovhus T., Rasmussen A., Bertoldo F., Larsen A. H., Mortensen J. J., Thygesen K. S. (2021). Comput. Mater. Sci..

[cit57] Mortensen J. J., Larsen A. H., Kuisma M., Ivanov A. V., Taghizadeh A., Peterson A., Haldar A., Dohn A. O., Schäfer C., Jónsson E. Ö. (2024). et al.. J. Chem. Phys..

[cit58] Enkovaara J. E., Rostgaard C., Mortensen J. J., Chen J., Dułak M., Ferrighi L., Gavnholt J., Glinsvad C., Haikola V., Hansen H. (2010). et al.. J. Phys.: Condens. Matter.

[cit59] Behler J. , Csányi G., Foppa L., Kang K., Langer M. F., Margraf J. T. *et al.*, Workflows for Artificial Intelligence, https://hdl.handle.net/21.11116/0000-0010-4C5A-5, 2024

[cit60] https://gitlab.com/taskblaster/taskblaster

[cit61] https://taskblaster.readthedocs.io/

